# Kynurenine levels are elevated in patients with heart failure with reduced ejection fraction or atrial fibrillation undergoing hemodialysis

**DOI:** 10.3892/mi.2026.313

**Published:** 2026-04-01

**Authors:** Maria Divani, Aikaterini Katsanaki, Panagiota Makri, Christina Poulianiti, Evangelos Lykotsetas, Andriani Balatsouka, Maria Tziastoudi, Ioannis Stefanidis, Theodoros Eleftheriadis

**Affiliations:** Department of Nephrology, Faculty of Medicine, University of Thessaly, 41110 Larissa, Greece

**Keywords:** kynurenine, inflammation, heart failure, atrial fibrillation, coronary heart disease, hemodialysis

## Abstract

Cardiovascular disease (CVD) is the leading cause of mortality in patients undergoing hemodialysis (HD). Uremic toxins may play a role in this process. One such toxin is kynurenine, a product of tryptophan metabolism that is not cleared sufficiently by HD. In the present study, serum kynurenine and tryptophan levels were measured using ELISA in 119 patients undergoing HD and 25 healthy subjects. In total, 41 patients had coronary heart disease (CHD), 61 patients had heart failure (HF), 27 patients had HF with reduced ejection fraction (HFrEF) and 33 patients had atrial fibrillation (AF). Factors that may influence kynurenine levels in patients undergoing HD, and the associations between kynurenine and CHD, HF and AF were examined. Compared with the healthy individuals, patients undergoing HD had higher kynurenine levels (7.56 vs. 2.33 µM; P<0.001). In patients undergoing HD, kynurenine negatively correlated with inflammatory markers. Kynurenine levels were significantly higher in patients undergoing HD with HFrEF (8.78 vs. 7.56 µM; P=0.03) and AF (9.15 vs. 7.30 µM; P=0.011). Following adjustment, for every 1 µM increase in serum kynurenine levels, the odds ratios for HFrEF and AF were 1.65 (P=0.008) and 1.37 (P=0.039), respectively. On the whole, the present study demonstrates that in patients undergoing HD, serum kynurenine levels are elevated, and are associated with HFrEF and AF. These findings suggest a potential role for this uremic toxin in the development of these cardiovascular disorders.

## Introduction

Cardiovascular disease (CVD) is the leading cause of mortality among patients undergoing hemodialysis (HD), with cardiovascular-related mortality being 10-20-fold higher patients undergoing HD than in the general population (1). Among the non-traditional factors that increase the risk of CVD in patients undergoing HD, are uremic toxins. Kynurenine is one such toxin. It is the initial molecule in the tryptophan breakdown through the kynurenine pathway. There are three reasons for kynurenine accumulation in patients undergoing HD. First, persistent inflammation, a characteristic of patients undergoing HD, increases indoleamine 2,3-dioxygenase (IDO)-1 expression in various tissues, mainly in monocytes, which then degrades tryptophan into kynurenine (2-4). The second enzyme that catalyzes tryptophan degradation to kynurenine is liver L-tryptophan 2,3-dioxygenase (TDO), and experimental data indicate that TDO activity increases in chronic kidney disease (CKD) (5). Finally, circulating kynurenine levels increase in patients undergoing HD due to reduced renal excretion (3,6). Notably, since kynurenine is a protein-bound toxin, HD or hemodiafiltration does not effectively lower its blood levels (3,7). Although an HD session may reduce the plasma free fraction of the kynurenine concentration by ~60%, 67% protein binding precludes the elimination of total kynurenine, which rapidly replenishes free kynurenine levels (7,8). As multiple factors affect circulating kynurenine levels, the use of the serum kynurenine-to-tryptophan ratio (KTR) as an indicator of IDO-1 activity has been challenged (9), particularly in patients undergoing HD (10). More precisely, in a previous study, in a cohort of 228 patients undergoing HD, plasma KTR and peripheral blood mononuclear cell IDO-1 mean fluorescence intensity (MFI) were higher than those in the healthy controls, but they did not correlate with each other. Only the IDO-1 MFI was associated with immune suppression, as evidenced by its negative correlation with skin induration diameter in tuberculin skin testing and its positive correlation with clinical infection (10). Once produced, kynurenine exerts its effects by activating the aryl hydrocarbon receptor (AhR), thereby altering multiple cellular functions (11-15).

In the general population, kynurenine pathway metabolites have been associated with the incidence of heart failure (HF) and atrial fibrillation (AF), as well as with worse outcomes in patients with existing HF (16-18). In patients undergoing HD, kynurenine pathway metabolites have been linked to inflammation and prevalent atherosclerotic CVD (19), carotid intima-media thickness (20,21), time to a new CV event (21), and a 1-year risk of mortality (22). However, other researchers have failed to find a connection between kynurenine pathway metabolites and new CV events or overall mortality (7). Of note, a recent study on patients with CKD not undergoing HD found that kynurenine levels were associated with both the prevalence and incidence of HF (23).

Further mechanistic studies are required to confirm the role of kynurenine in CKD-related complications. Nonetheless, some data already exist. In a previous study on 116 patients with CKD stage 3-5D, the sera of the patients exhibited potent AhR-activating potential compared with the healthy controls (24). Additionally, the expression of the AhR target gene, CYP1A1, was upregulated in whole blood from patients with CKD. Survival analysis revealed that cardiovascular events were more common in patients with CKD with AhR-activating potential above the median (24). Another study analyzed samples from two large multicenter studies; one on patients with end-stage kidney disease and another on patients with CKD stage 2-3(25). In the first study, patients with arteriovenous fistula thrombosis had elevated kynurenine levels and AhR activity. In the second study, patients with postangioplasty thrombosis had higher kynurenine levels and AhR-activating potential. Mechanistic analyses *in vivo* revealed that kynurenine promotes thrombosis following vascular injury in an animal model and exerts its effects via AhR pathways. In primary human vascular smooth muscle cell cultures, kynurenine elevated AhR activity and tissue factor expression (25).

Considering the critical role of CVD in the mortality and morbidity of patients undergoing HD, it is crucial to investigate the underlying pathophysiological pathways responsible for CVD and, if possible, identify biomarkers for the early detection of CVD in this population. Studies conducted in patients undergoing HD have assessed kynurenine levels in relation to prevalent atherosclerotic disease, defined collectively as coronary heart disease (CHD), stroke, or peripheral arterial disease (19,20); incident atherosclerotic events and heart failure (21); incident atherosclerotic disease and all-cause mortality (7); or overall mortality (22). However, although CVDs have overlapping pathogenic pathways, these mechanisms are not completely identical. Therefore, the present study examined whether serum kynurenine levels are independently associated with prevalent CHD, HF or AF among patients undergoing HD. To the best of our knowledge, this is the first study to assess the association of kynurenine with prevalent HF and AF among patients undergoing HD.

## Patients and methods

### Patients

A total of 119 patients undergoing HD participated in the present study. The mean age of the patients was 65.98±11.87 years, including 87 males and 32 females. The causes of end-stage kidney disease were diabetic nephropathy (n=29), hypertension (n=24), primary glomerulonephritis (n=22), cardiorenal syndrome (n=7), autosomal dominant polycystic kidney disease (n=6), secondary focal segmental glomerulosclerosis (n=6), vasculitis (n=4), obstructive nephropathy (n=2), analgesic nephropathy (n=1) and unknown (n=18).

In total, 41 patients had diabetes mellitus, 103 patients had hypertension, 41 patients had a history of CHD, 33 patients had AF, and, according to a recent, within-3-months, transthoracic echocardiogram, 34 patients were diagnosed with HF with preserved ejection fraction (HFpEF), while 27 patients were diagnosed with HF with reduced ejection fraction (HFrEF). CHD was ascertained by coronary artery angiography performed for angina symptoms or after a myocardial infarction. The majority of patients received anti-hypertensive medications, and statins were prescribed to 79 of these patients. All patients with CHD were on clopidogrel or aspirin, along with a β-blocker. Patients with HFrEF received a β-blocker and a low-dose angiotensin receptor blocker. Those with AF were prescribed a β-blocker and anticoagulation. The policy of the HD unit of the University of Thessaly is to avoid anti-inflammatory medications. The attending nephrologists determined the need for phosphate binders, vitamin D analogs, calcimimetics, erythropoietin and intravenous iron. All patients had been undergoing HD for at least 6 months prior to inclusion. Each patient underwent 4-h HD sessions, three times a week, using polysulfone dialyzers and bicarbonate-based dialysate with calcium concentrations of either 1.25 or 1.5 mmol/l.

The exclusion criteria included active infection, autoimmune disease, malignancy, liver pathology, or treatment with cytotoxic, immunosuppressive, or corticosteroid medications within the prior 6 months. The clinical, laboratory and demographic features of the patients undergoing HD are presented in Table I.

A control group of 25 healthy individuals (mean age, 64.60±6.69 years; 16 males and 9 females) was included following a review of their medical records and a physical examination.

Written informed consent was obtained from each participant, and the study protocol was approved by the Ethics Committee of the Faculty of Medicine, University of Thessaly, Larissa, Greece (approval no. 558/10-2-2017).

### Sample collection

Blood samples were collected at the beginning of the second HD session of the week, and serum was stored at -80˚C. Serum total kynurenine levels were measured using the Kynurenine ELISA kit (cat. no. KA6140, Abnova Corporation), which has a detection limit of 0.22 µM and a calibration range of 0.30-48 µM. Serum total tryptophan levels were measured using the Tryptophan ELISA kit (cat. no. BA E-2700, Labor Diagnostika Nord GmbH & CoKG), which has a detection limit of 3.18 µM and a calibration range of 0-1223 µM. All other parameters were part of routine laboratory assessments conducted alongside serum collection for kynurenine and tryptophan measurement.

### Statistical analysis

Statistical analysis was performed using IBM SPSS Statistics version 29 (IBM Corp.) and JASP version 0.95.4 (University of Amsterdam, Amsterdam, The Netherlands). As the one-sample Kolmogorov-Smirnov test revealed that the analyzed variables were not normally distributed, non-parametric methods were employed. Specifically, for group comparisons, the Mann-Whitney U test was used, with data presented as the median (interquartile range). A post hoc power analysis for the kynurenine-related Mann-Whitney U test was performed using the observed rank-biserial correlation (r), group sample sizes, and a two-tailed significance level of α=0.05 to estimate achieved statistical power under the nonparametric framework. Additionally, Spearman's Rho was calculated to assess correlations between continuous variables, while the Chi-squared test, with Fisher's exact test when needed, were applied to evaluate the associations between categorical variables. Receiver operating characteristic (ROC) curve analysis was performed to assess the diagnostic utility of kynurenine. However, as the pathogenesis of the evaluated diseases is multifactorial, a single factor is unlikely to achieve good discrimination (AUC >0.8). Therefore, the possible pathogenetic role of kynurenine was evaluated using binary logistic regression, including all factors associated with the evaluated diseases. Multicollinearity among predictors was evaluated using the variance inflation factor (VIF). Given the small sample size, VIF values <2.5 were considered acceptable. To assess whether kynurenine enhances discrimination of the evaluated diseases, along with other confounding factors, the model fit was re-evaluated using the change of Χ^2^ (ΔΧ^2^) and its statistical significance, as well as AUC and Nagelkerke R^2^, for each logistic regression analysis after removing kynurenine from the model. A value of P<0.05 was considered to indicate a statistically significant difference.

## Results

### Kynurenine levels in healthy individuals and in patients undergoing HD

Serum kynurenine levels were significantly higher in patients undergoing HD than in the healthy individuals [7.56 (3.26) µM vs. 2.33 (1.32) µM, respectively; P<0.001] (Table II). A post hoc power analysis was conducted using the observed effect size derived from the Mann-Whitney U test (r=0.989; approximated Cohen's d=5.34), α=0.05, and the achieved sample sizes (n_1_=25, n_2_=119). The calculated power was 1.00 (Table III).

Notably, serum tryptophan levels were significantly lower in patients undergoing HD than in the healthy individuals [31.17 (18.67) µM vs. 53.66 (18.87) µM, respectively; P<0.001]. The KTR was higher in patients undergoing HD [0.235 (0.143) vs. 0.05 (0.027); P<0.001] (Table II).

In patients undergoing HD, sex, diabetes mellitus, hypertension, CHD and HF as a whole did not affect kynurenine levels. However, kynurenine levels were higher in patients undergoing HD with HFrEF [8.78 (3.74) Μm vs. 7.56 (3.32) µM; P=0.030] (Table II). A post hoc power analysis was conducted using the observed effect size derived from the Mann-Whitney U test (r=0.275; approximated Cohen's d=0.57), α=0.05, and the achieved sample sizes (n_1_=27, n_2_=92) (Table III). The calculated power was 0.77. Kynurenine levels were also higher in patients with AF [9.15 (3.57) µM vs. 7.30 (3.34) µM; P=0.011] (Table II). A post hoc power analysis was conducted using the observed effect size derived from the Mann-Whitney U test (r=0.303; approximated Cohen's d=0.64), α=0.05, and the achieved sample sizes (n_1_=33, n_2_=86). The calculated power was 0.88 (Table III).

In patients with C-reactive protein (CRP) levels >1 mg/dl, serum kynurenine levels were lower [6.68 (4.13) µM vs. 8.08 (2.70) µM; P=0.024]. Notably, serum tryptophan levels did not differ between patients with or without HFrEF [28.48 (20.14) µM vs. 31.33 (18.6) µM, respectively; P=0.741] or between patients with or without AF [28.83 (18.68) µM vs. 31.90 (18.58) µM, respectively; P=0.621]. KTR did not differ between patients undergoing HD with or without HFrEF [0.246 (0.194) vs. 0.231 (0.121), respectively; P=0.201]. However, KTR was higher in patients undergoing HD with AF [0.254 (0.158) vs. 0.226 (0.119); P=0.024] (Table II).

**Figure 1:**
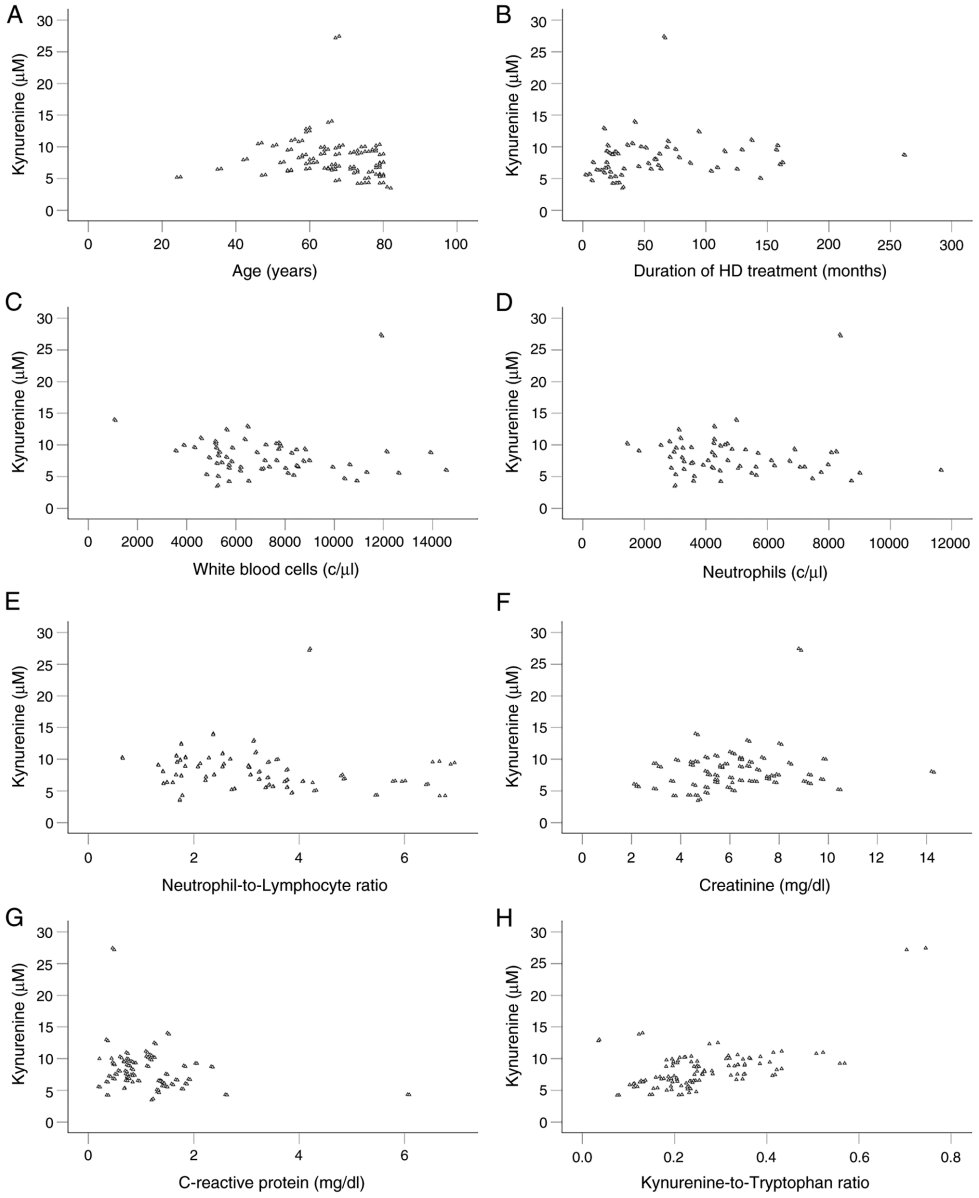
Scatterplots of statistically significant correlations of kynurenine with other variables in patients undergoing hemodialysis. Scatterplots depicting kynurenine in relation to (A) age, (B) the duration of hemodialysis treatment, (C) white blood cell count, (D) neutrophil count, (E) neutrophil-to-lymphocyte ratio, (F) creatinine levels, (G) C-reactive protein, and (H) the kynurenine-to-tryptophan ratio.

Among the factors analyzed in the present study and shown in Table I, serum kynurenine levels correlated with age (Rho=-0.245, P=0.007), the duration of HD (Rho=0.330, P<0.001), white blood cell count (Rho=-0.255, P=0.005), neutrophil count (Rho=-0.228, P=0.013), neutrophil-to-lymphocyte ratio (Rho=-0.265, P=0.004), creatinine (Rho=0.183, P=0.046) and CRP (Rho=-0.268, P=0.003). Notably, kynurenine did not correlate with tryptophan (Rho=0.143, P=0.126), whereas a positive correlation was observed with KRT (Rho=0.486, P<0.001) (Table IV). Scatterplots illustrating kynurenine and its statistically significant correlated factors are presented in Fig. 1.

### Kynurenine as a determinant of HFrEF in patients undergoing HD

In addition to elevated kynurenine levels, patients undergoing HD with HFrEF were on HD treatment for a longer period of time [66 (54.0) months vs. 32 (49.5) months; P=0.034], had an elevated neutrophil-to-lymphocyte ratio [3.600 (1.713) vs. 2.706 (1.725); P=0.026] and had lower triglyceride levels [87.0 (48.0) vs. 114.5 (78.5) mg/dl; P=0.012]. HFrEF was associated with the male sex (Pearson χ^2^=6.743, P=0.009) and CHD (Pearson χ^2^=29.027, P<0.001) (Table V).

**Figure 2:**
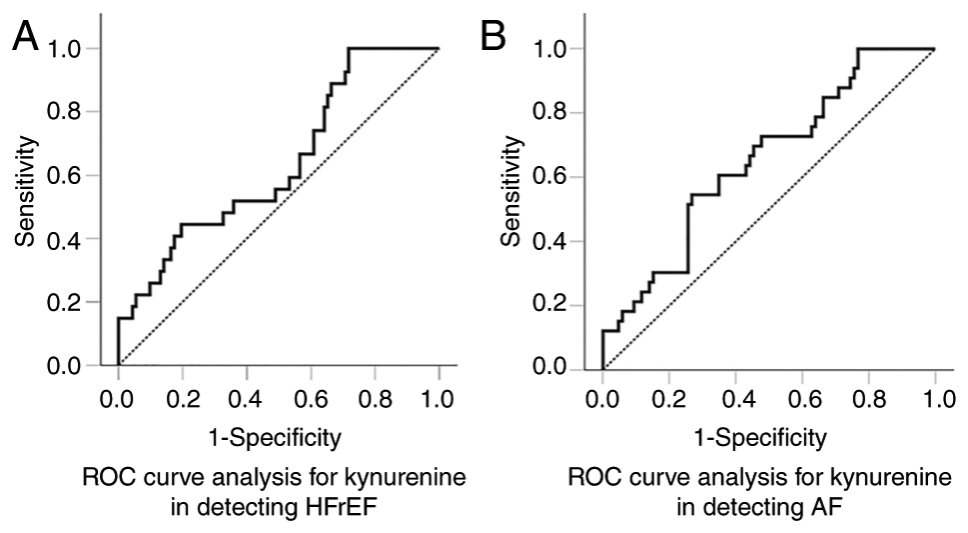
ROC curve analysis evaluating the ability of kynurenine to detect HFrEF and AF. (A) ROC curve analysis revealed a modest discriminative ability of kynurenine for HFrEF, with an AUC of 0.637 (95% CI, 0.520-0.754; P=0.022). (B) Similarly, ROC curve analysis demonstrated a modest discriminative ability of kynurenine for AF, with an AUC of 0.652 (95% CI, 0.545-0.758; P=0.005). ROC, receiver operating characteristic; HFrEF, heart failure with reduced ejection fraction; AF, atrial fibrillation.

ROC curve analysis revealed a modest discriminative ability of kynurenine for HFrEF, with an AUC of 0.637 [95% confidence interval (CI), 0.520-0.754; P=0.022] (Fig. 2A). At the optimal cut-off of 6.11 µM, the test had 100% sensitivity and 28.3% specificity, indicating excellent rule-out capability but poor rule-in performance. Binary logistic regression, including kynurenine and other factors linked to HFrEF, as demonstrated in in Table VI, indicated that kynurenine was a significant predictor of HFrEF in patients undergoing HD. For every 1 µM increase in the serum kynurenine level, the odds of HFrEF increased by 65% [odds ratio (OR), 1.649; 95% CI, 1.141-2.381; P=0.008] (Table VI). Removing kynurenine from the model decreased model fit (Χ^2^ decreases, ΔΧ^2^=20.722, P<0.001), the AUC from 0.904 to 0.859, and Nagelkerke R^2^ from 0.581 to 0.449. Thus, kynurenine is unlikely to serve as a standalone diagnostic marker for HFrEF, reflecting the multifactorial nature of the disease; however, it remains a risk factor that may contribute to its pathogenesis.

### Kynurenine as a determinant of AF in patients undergoing HD

In addition to elevated kynurenine levels, patients with AF undergoing HD were older [72 (13.5) years vs. 66 (18.0) years; P=0.033], had an elevated white blood cell count [8,300 (2,245.0) vs. 6,220 (2,802.5) c/µl; P=0.006], neutrophil count [5647.0 (2244.0) vs. 4194.5 (1868.5) c/µl; P<0.001] and neutrophil-to-lymphocyte ratio [3.17 (2.06) vs. 2.72 (1.96); P=0.024]. In addition, they had lower hemoglobin [11.40 (2.30) vs. 11.95 (1.20) g/dl, P=0.003], lower phosphorus [4.8 (1.28) vs. 5.2 (1.53) mg/dl; P=0.030] and higher ferritin [164.4 (258.15) vs. 106.9 (129.7) ng/ml, P=0.043] levels, and a lower transferrin saturation [14.95 (9.11) vs. 17.13 (10.99)%; P=0.042]. As noted, KTR was higher in patients with AF undergoing HD (Table VII).

ROC curve analysis demonstrated a modest discriminative ability of kynurenine for AF, with an AUC of 0.652 (95% CI, 0.545-0.758; P=0.005) (Fig. 2B). At the optimal cut-off value of 9.0 µM, sensitivity was 54.5%, and specificity was 73.3%, indicating moderate diagnostic performance without strong rule-in or rule-out. Binary logistic regression, including kynurenine and other factors linked to AF, demonstrated in Table VIII, revealed that kynurenine was a significant predictor of AF in patients undergoing HD. For each 1 µM increase in serum kynurenine level, the odds of AF increase by 37% (OR, 1.372; 95% CI, 1.016-1.853; P=0.039) (Table VIII). Removing kynurenine and KTR from the model decreased model fit (Χ^2^ decreases, ΔΧ^2^=13.817, P<0.001), the AUC from 0.871 to 0.814, and Nagelkerke R^2^ from 0.498 to 0.371. Thus, kynurenine is unlikely to serve as a standalone diagnostic marker for AF, which is consistent with the multifactorial nature of the disease; however, it appears to be a risk factor that may contribute to the pathogenesis of AF.

## Discussion

Considering the high CV morbidity and mortality rates in patients undergoing HD (1), the present study investigated the impact of the uremic toxin kynurenine on CVD by measuring its serum levels in patients undergoing HD with or without CHD, HF or AF.

As expected, an increased kynurenine production (2-5), reduced renal elimination (3,6) and the inability of HD to achieve sufficient kynurenine clearance (3,7) led to significantly higher kynurenine levels in patients undergoing HD compared to healthy volunteers. Notably, although previous studies have reported a positive correlation between kynurenine pathway metabolites and inflammation (3,19,20), others have failed to detect such an association (6,7). The present study observed a negative correlation between serum kynurenine and CRP or the neutrophil-to-lymphocyte ratio. IDO-1 is present in various cell types and, upon upregulation by inflammatory stimuli, exerts immunosuppressive and anti-inflammatory effects (26,27). In the immune microenvironment, the upregulation of IDO-1 in monocytes leads to local tryptophan depletion and kynurenine production, which activate general control nonderepressible-2 kinase and AhR, respectively. As a result, cell metabolism shifts to suppress the adaptive immune response by reducing T-cell proliferation and favoring CD4^+^ T-cell differentiation toward regulatory rather than effector phenotypes (14,28-30). As regards natural killer cells, IDO-1 impairs their function by downregulating NKG2D ligand via A disintegrin and metalloproteinase domain-containing protein 10(31). As regards neutrophils, IDO-1 activity limits neutrophil abundance and pro-inflammatory function (32,33). In epithelial cells, IDO-1 is upregulated following viral infection and subsequently downregulates NF-κB signaling and the production of the pro-inflammatory cytokines, IFN-α, IFN-β and IL-6(34). Of note, in antigen-presenting cells, AhR activation is required for IDO-1 expression, suggesting a positive feedback loop that may enhance IDO-1 immunosuppressive properties (35). It is likely that the negative correlation between serum kynurenine levels and the inflammatory markers CRP and neutrophil-to-lymphocyte ratio detected in the present study reflects IDO-1 upregulation due to chronic inflammation that characterizes patients undergoing HD, which subsequently counteracts inflammation. Notably, compared with healthy controls, IDO-1 expression is higher in monocytes from patients undergoing HD (4). Remarkably, in a previous study, in a group of stable patients undergoing HD, plasma IDO-1 levels were higher than those in healthy subjects and negatively correlated with CRP, IL-6 and TNF-α (36).

In the present study, serum kynurenine levels did not differ between patients undergoing HD with or without CHD. Previous studies have identified a link between kynurenine pathway metabolites and subclinical atherosclerosis, prevalent atherosclerotic CVD, new CV events, and mortality in patients undergoing HD (19-22). However, in a cohort of patients undergoing HD from the CONTRAST trial with a median follow-up of 4.3 years, baseline kynurenine pathway metabolite levels were not associated with all-cause mortality or new CV events (7). Differences in the characteristics of the patients across these studies may account for this discrepancy.

In the general population, kynurenine pathway metabolites have been linked to the occurrence of HF and AF, as well as to worse outcomes in patients with existing HF (16-18). In patients with CKD, the prevalence of HF is high and is associated with increased mortality (37). In a previous study, in a cohort of 673 non-dialysis patients with CKD stages 1-5, kynurenine levels were connected to both prevalent and incident HF (23). The present study assessed whether kynurenine levels varied between patients with and without HF undergoing HD. It was found that serum kynurenine was elevated only in patients with HFrEF. ROC curve analysis demonstrated a modest discriminatory ability of kynurenine as a biomarker for HFrEF in patients undergoing HD, with an AUC of 0.637 (P=0.022). However, given that HFrEF is a multifactorial disorder, a single variable is unlikely to provide strong diagnostic accuracy. Therefore, a binary logistic regression analysis was performed to determine whether kynurenine is an independent risk factor and a potential pathogenetic contributor to HFrEF in this population. After adjusting for other factors associated with HFrEF, kynurenine remained an independent predictor. For each 1 µM increase in serum kynurenine level, the odds of HFrEF increased by 65%. Notably, in the patient cohort in the present study, 21 of the 27 patients with HFrEF also had CHD. Experimental data support the role of kynurenine pathway products in the development of HF. Following myocardial infarction, IDO-1 is upregulated in endothelial cells, leading to increased kynurenine production. This, through the AhR pathway, causes cardiomyocyte apoptosis (38). An elevated expression of IDO-1 has been observed in the hypertrophic myocardium, and increased kynurenine activates the AhR, leading to cardiac hypertrophy (39). Finally, monocyte infiltration plays a significant role in cardiac fibrosis (40), and in patients undergoing HD, monocytes express higher levels of IDO-1(4).

The present study then examined whether kynurenine levels vary between patients with and without AF undergoing HD. In patients undergoing HD, the prevalence of AF is higher than that in the general population and is linked to increased mortality (41). Even following effective treatment of AF with pulmonary vein isolation (42,43), the recurrence rate is significantly higher in patients undergoing HD, indicating the vulnerability of this population to AF (44,45). The present study found that serum kynurenine levels were higher in patients with AF. ROC curve analysis revealed the modest performance of kynurenine as a biomarker for AF in patients undergoing HD, with an AUC of 0.652 (P=0.005). However, for a multifactorial condition such as AF, a single factor is unlikely to achieve good discrimination. Thus, binary logistic regression analysis was used to evaluate whether kynurenine is an independent risk factor and possibly a pathogenetic factor for AF in this population. After adjusting for other factors associated with AF, kynurenine remained an independent predictor. For each 1 µM increase in serum kynurenine, the odds of AF increased by 37%. Whether this is caused by cardiac fibrosis or a direct arrhythmogenic effect of kynurenine pathway products remains to be clarified. Experimentally, kynurenine exerts pro-arrhythmogenic effects by modulating cardiac repolarization, possibly by reducing Kv11.1 channel levels (46). In other cell types, an increased expression of IDO-1 has been shown to upregulate P2X7 purinoceptor expression (47). Experimentally, P2X7 predisposes to AF by altering ion channel protein expression and inducing myocardial hypertrophy and fibrosis (48,49).

To the best of our knowledge, this is the first study to assess the role of kynurenine in HF and AF among patients undergoing HD. However, there are some limitations. Although our study detected an association between serum kynurenine levels and the prevalence of HFrEF and AF, its cross-sectional design cannot establish causality. Future studies that measure kynurenine longitudinally could clarify causal associations and may help establish serum kynurenine as a risk factor for HFrEF and AF among patients undergoing HD. For this reason, the authors plan to follow-up with the patients and enroll additional patients. In addition, the enrollment of patients from other centers will verify the stability of the association between kynurenine and cardiovascular complications. Another limitation of the present study is the inclusion of patients with a known history of CHD, which may have led to an underestimation of the true prevalence of CHD, as no specific diagnostic imaging was performed to detect clinically silent cases. Nevertheless, patients undergoing HD attend the HD unit three times per week, facilitating the early detection of CHD symptoms. Furthermore, these patients receive regular cardiological assessments and transthoracic echocardiography. As regards HF, all patients underwent a transthoracic echocardiogram within 3 months of the study. AF was unlikely to remain undiagnosed, given the frequency of patient visits to the HD unit.

In the event that the contribution of kynurenine to the development of HF or AF in patients undergoing HD is confirmed, strategies to reduce its levels may warrant consideration. As IDO-1 significantly contributes to kynurenine production, reducing its expression by alleviating the chronic inflammation typical of HD is a viable approach. Notably, clinical trials, such as the large phase III clinical trials NCT05021835 and the NCT05485961, are already underway using monoclonal antibodies targeting pro-inflammatory cytokines to reduce chronic inflammation (50). Alternatively, the direct inhibition of IDO-1 is possible. Several specific IDO-1 inhibitors, as well as inhibitors of both IDO-1 and TDO, are available and have been shown to be safe, having been tested as immunomodulatory agents in cancer treatment (51).

In conclusion, as demonstrated in the present study, in patients undergoing HD, serum kynurenine levels are elevated and associated with HFrEF and AF, suggesting a potential role for this uremic toxin in the development of these CV disorders. If proven, efforts to decrease tryptophan metabolism via the kynurenine pathway by reducing inflammation or inhibiting IDO-1 would be beneficial, as removing already formed kynurenine pathway products is challenging due to their binding to serum proteins.

## Figures and Tables

**Table I:** Characteristics of the patients undergoing hemodialysis.

Characteristic	N	Median	25-75% percentile
Age (years)	119	67	59-76
Duration of HD treatment (months)	119	42	22-42
Males/females	87/32		
Diabetes mellitus (yes/no)	44/75		
Hypertension (yes/no)	103/14		
Coronary heart disease (yes/no)	41/78		
HFrEF/HFpEF/no HF	27/34/58		
Atrial fibrillation (yes/no)	33/86		
White blood cell count (c/µl)	119	7,800	5,360-8,500
Neutrophils (c/µl)	119	4,310	3,277-6,120
Lymphocytes (c/µl)	119	1,725	1,236-2,064
Neutrophil-to-lymphocyte ratio	119	3.039	1.839-3.778
Hemoglobin (g/dl)	119	11.7	11.2-12.4
Platelets (c/µl)	119	199,000	162,000-242,000
Creatinine (mg/dl)	119	6.0	4.7-7.5
Urea (mg/dl)	119	122	107-144
Urea reduction rate (%)	119	67.797	63.115-72.727
Residual diuresis (ml)	119	0	0-600
Body mass index (kg/m²)	119	25.333	22.773-29.201
Protein catabolic rate (g/kg/day)	119	0.880	0.715-0.990
Albumin (g/dl)	119	3.6	3.5-3.8
Cholesterol (mg/dl)	119	136.0	100.0-153.0
Triglyceride (mg/dl)	119	109.0	82.0-167.0
Calcium (mg/dl)	119	9.1	8.9-9.5
Phosphorous (mg/dl)	119	5.1	4.5-5.9
Parathyroid hormone (pg/ml)	119	240.81	155.61-471.80
Alkaline phosphatase (U/l)	119	186.0	147.0-224.0
SGOT (U/l)	119	12.0	9.0-15.0
SGPT (U/l)	119	9.0	6.0-15.0
Ferritin (ng/ml)	119	120	58.9-228.4
TSAT (%)	119	16.748	12.136-22.434
CRP (mg/dl)	119	0.88	0.69-1.4
CRP >1 mg/dl (yes/no)	56/63		
Kynurenine (µM)	119	7.562	6.317-9.572
Tryptophan (µM)	119	31.17	26.27-44.56
Kynurenine-to-tryptophan ratio	119	0.235	0.187-0.326

HD, hemodialysis; HFrEF, heart failure with reduced ejection fraction; HFpEF, heart failure with preserved ejection fraction; HF, heart failure; SGOT, serum glutamic oxaloacetic transaminase; SGPT, serum glutamic pyruvic transaminase; TSAT, transferrin saturation; CRP, C-reactive protein.

**Table II:** Kynurenine, tryptophan and kynurenine-to-tryptophan ratio in healthy controls and patients undergoing hemodialysis, and subgroups of patients undergoing hemodialysis.

Comparison	Kynurenine (µM)	P-value	Tryptophan (µM)	P-value	Kynurenine-to-tryptophan ratio	P-value
HD patients vs. healthy controls	7.56 (3.26) vs. 2.33 (1.32)	**<0.001**	31.17 (18.67) vs. 53.66 (18.87)	**<0.001**	(0.235 (0.143) vs. 0.05 (0.027)	**<0.001**
Patients undergoing HD						
Males vs. females	7.53 (2.84) vs. 8.34 (4.27)	0.801	31.49 (15.83) vs. 30.39 (28.81)	0.584	0.239 (0.127) vs. 0.218 (0.189)	0.540
Diabetes mellitus, yes vs. no	6.91 (3.86) vs. 7.60 (3.43)	0.062	31.79 (19.74) vs. 30.83 (17.97)	0.916	0.206 (0.167) vs. 0.242 (0.139)	0.079
Hypertension, yes vs. no	7.60 (3.15) vs. 7.23 (3.10)	0.271	30.83 (19.39) vs. 34.85 (8.07)	0.312	0.236 (0.154) vs. 0.207 (0.064)	0.210
Coronary heart disease, yes vs. no	6.87 (3.60) vs. 8.05 (3.14)	0.345	36.86(19.99) vs. 28.62 (15.83)	**0.001**	0.194 (0.095) vs. 0.249 (0.126)	**<0.001**
Heart failure, yes vs. no	7.37 (3.53) vs. 7.62 (3.53)	0.312	32.35 (18.50) vs. 29.82 (18.71)	0.780	0.232 (0.147) vs. 0.242 (0.143)	0.951
HFrEF, yes vs. no	8.78 (3.74) vs. 7.56 (3.32)	**0.030**	28.48 (20.14) vs. 31.33 (18.6)	0.741	0.246 (0.194) vs. 0.231 (0.121)	0.201
Atrial fibrillation, yes vs. no	9.15 (3.57) vs. 7.30 (3.34)	**0.011**	28.83 (18.68) vs. 31.90 (18.58)	0.621	0.254 (0.158) vs. 0.226 (0.119)	**0.024**
CRP >1 mg/dl, yes vs. no	6.68 (4.13) vs. 8.08 (2.70)	**0.024**	29.55 (18.98) vs. 32.08 (18.15)	0.560	0.228 (0.060) vs. 0.249 (0.182)	0.163

Values in bold font indicate statistically significant differences (P<0.05). HD, hemodialysis; HFrEF, heart failure with reduced ejection fraction; CRP, C-reactive protein.

**Table III:** Post hoc power analyses for kynurenine-related Mann-Whitney U tests.

Comparison	Observed effect size (r)	Cohen's d (approx.)	Sample size (n)	Significance level (α)	Post hoc power (1-β)
Kynurenine: Controls vs. patients undergoing HD	0.989	5.34	Control: 25	0.05	1.00
			HD patients: 119		
Kynurenine: HFrEF vs. no HFrEF	0.275	0.57	HFrEF: 27	0.05	0.77
			No HFrEF: 92		
Kynurenine: AF vs. no AF	0.303	0.64	AF: 33	0.05	0.88
			No AF: 86		

HD, hemodialysis; HFrEF, heart failure with reduced ejection fraction; AF, atrial fibrillation.

**Table IV:** Correlations of kynurenine with other variables in patients undergoing hemodialysis.

	Rho	P-value
Age	-0.245	**0.007**
Duration of HD treatment	0.330	**<0.001**
White blood cells	-0.255	**0.005**
Neutrophils	-0.228	**0.013**
Lymphocytes	0.070	0.451
Neutrophil-to-Lymphocyte ratio	-0.265	**0.004**
Hemoglobin	-0.128	0.165
Platelets	-0.110	0.235
Creatinine	0.183	**0.046**
Urea	0.034	0.714
Urea reduction ratio	-0.021	0.863
Residual diuresis	-0.008	0.934
Body mass index	0.100	0.277
Protein catabolic ratio	0.176	0.055
Albumin	0.100	0.281
Cholesterol	-0.172	0.061
Triglycerides	0.071	0.442
Calcium	0.045	0.625
Phosphoros	-0.152	0.099
Parathyroid hormone	0.142	0.122
Alkaline phosphatase	0.125	0.176
SGOT	0.059	0.521
SGPT	0.114	0.216
Ferritin	0.086	0.353
Transferrin saturation	-0.109	0.239
C-reactive protein	-0.268	**0.003**
Tryptophan	0.143	0.126
Kynurenine-to-tryptophan ratio	0.486	**<0.001**

Values in bold font indicate statistically significant differences (P<0.05). HD, hemodialysis; SGOT, serum glutamic oxaloacetic transaminase; SGPT, serum glutamic pyruvic transaminase.

**Table V:** Characteristics of patients undergoing hemodialysis with and without heart failure with a reduced ejection fraction.

	No HFrEF (n=92)	HFrEF (n=27)	P-value
Age (years)	67 (16.75)	67 (15.0)	0.706
Duration of HD treatment (months)	32 (49.5)	66 (54.0)	**0.034**
Males/females	62/30	25/2	**0.009**
Diabetes mellitus (yes/no)	36/56	8/19	0.369
Hypertension (yes/no)	78/12	25/2	0.517
Coronary heart disease (yes/no)	20/72	21/6	**<0.001**
Atrial fibrillation (yes/no)	22/70	11/16	0.086
White blood cell count (c/µl)	7,205 (2,817.5)	6,890 (3,980.0)	0.146
Neutrophils (c/µl)	4,379.5 (2,369.3)	4,226 (3,472.0)	0.814
Lymphocytes (c/µl)	1,790(703)	1,645(974)	0.075
Neutrophil-to-lymphocyte ratio	2.706 (1.725)	3.600 (1.713)	**0.026**
Hemoglobin (g/dl)	11.80 (1.20)	11.70 (1.40)	0.228
Platelets (c/µl)	199,000 (74,500)	202,000 (117,000)	0.914
Creatinine (mg/dl)	5.90 (2.97)	6.10 (1.70)	0.671
Urea (mg/dl)	122.5 (34.5)	122.0 (37.0)	0.542
Urea reduction rate (%)	67.93 (9.75)	67.68 (9.43)	0.785
Residual diuresis (ml)	0(600)	300(600)	0.464
Body mass index (kg/m^2^)	25.183 (6.29)	25.432 (6.07)	0.310
Protein catabolic rate (g/kg/day)	0.852 (0.263)	0.926 (0.229)	0.207
Albumin (g/dl)	3.65 (0.3)	3.60 (0.2)	0.336
Cholesterol (mg/dl)	139 (53.5)	124 (55.0)	0.050
Triglyceride (mg/dl)	114.5 (78.5)	87.0 (48.0)	**0.012**
Calcium (mg/dl)	9.1 (0.4)	9.2 (1.2)	0.506
Phosphorous (mg/dl)	5.1 (1.47)	5.2 (1.30)	0.295
Parathyroid hormone (pg/ml)	285.66 (300.4)	208.31 (238.5)	0.243
Alkaline phosphatase (U/l)	192 (97.5)	179 (57.0)	0.650
SGOT (U/l)	12 (6.75)	12 (4.00)	0.944
SGPT (U/l)	9(8)	9(11)	0.325
Ferritin (ng/ml)	114.3 (145.82)	141.1 (254.60)	0.542
Transferrin saturation (%)	17.17 (11.82)	14.52 (4.82)	0.129
CRP (mg/dl)	1.0 (0.72)	0.85 (0.71)	0.854
CRP >1 mg/dl (yes/no)	46/46	10/17	0.235
Kynurenine (µM)	7.52 (3.41)	8.78 (3.74)	**0.030**
Tryptophan (µM)	31.33 (18.6)	28.48 (20.14)	0.741
Kynurenine-to-tryptophan ratio	0.231 (0.121)	0.246 (0.194)	0.201

Values in bold font indicate statistically significant differences (P<0.05). HD, hemodialysis; HFrEF, heart failure with reduced ejection fraction; SGOT, serum glutamic oxaloacetic transaminase; SGPT, serum glutamic pyruvic transaminase.

**Table VI:** Binary logistic regression analysis of factors associated with HFrEF.

					95% CI for OR		
Comparison	B	S.E.	Sig.	OR	Lower	Upper	VIF
Duration of HD treatment	0.003	0.006	0.642	1.003	0.991	1.014	1.263
Female sex	-0.105	0.954	0.913	0.901	0.139	5.838	1.295
Coronary heart disease	3.535	0.839	**<0.001**	34.312	6.628	177.628	1.864
Neutrophile-to-lymphocyte ratio	0.388	0.251	0.122	1.474	0.901	2.412	1.526
Triglycerides (per mg/dl)	-0.022	0.007	**0.003**	0.978	0.965	0.992	1.380
Kynurenine (per µM)	0.500	0.188	**0.008**	1.649	1.141	2.381	1.809

Model fit: Nagelkerke R^2^ = 0.581, AUC=0.904, model fit: X^2^=57.254, P<0.001. Values in bold font indicate statistically significant differences (P<0.05). HD, hemodialysis; HFrEF, heart failure with reduced ejection fraction.

**Table VII:** Characteristics of hemodialysis patients with and without atrial fibrillation.

Characteristic	No Atrial fibrillation (n=86)	Atrial fibrillation (n=33)	P-value
Age (years)	66 (18.0)	72 (13.5)	**0.033**
Duration of HD treatment (months)	33.5 (54.25)	48.0 (79.00)	0.276
Males/females	26/60	6/27	0.184
Diabetes mellitus (yes/no)	30/56	14/19	0.446
Hypertension (yes/no)	74/10	29/4	1.000
Coronary heart disease (yes/no)	28/58	13/20	0.482
HFrEF (yes/no)	16/70	11/22	0.086
White blood cell count (c/µl)	6,220 (2,802.5)	8,300 (2,245.0)	**0.006**
Neutrophils (c/µl)	4,194.5 (1,868.5)	5,647.0 (2,244.0)	**<0.001**
Lymphocytes (c/µl)	1,678.5 (841.5)	1,821.0 (709.0)	0.549
Neutrophil-to-lymphocyte ratio	2.72 (1.96)	3.17 (2.06)	**0.024**
Hemoglobin (g/dl)	11.95 (1.20)	11.40 (2.30)	**0.003**
Platelets (c/µl)	200500 (77250)	196000 (92000)	0.523
Creatinine (mg/dl)	6.10 (2.72)	5.80 (3.20)	0.553
Urea (mg/dl)	122.5 (39.5)	122.0 (35.5)	0.722
Urea reduction rate (%)	68.19 (10.50)	67.33 (7.14)	0.854
Residual diuresis (ml)	0(700)	0(500)	0.449
Body mass index (kg/m^2^)	25.24 (6.35)	26.81 (6.53)	0.254
Protein catabolic rate (g/kg/day)	0.848 (0.268)	0.898 (0.252)	0.219
Albumin (g/dl)	3.7 (0.3)	3.6 (0.2)	0.073
Cholesterol (mg/dl)	137.5 (46.5)	124.0 (66.0)	0.157
Triglyceride (mg/dl)	109.5 (85.5)	109.0 (98.5)	0.856
Calcium (mg/dl)	9.1 (0.58)	9.1 (0.8)	0.260
Phosphorus (mg/dl)	5.2 (1.53)	4.8 (1.28)	**0.030**
Parathyroid hormone (pg/ml)	240.31 (196.9)	300.10 (411.2)	0.652
Alkaline phosphatase (U/l)	175.0 (82.5)	198.0 (57.5)	0.854
SGOT (U/l)	12.0 (6.0)	13.0 (4.5)	0.128
SGPT (U/l)	9.0 (9.25)	10.0 (8.5)	0.793
Ferritin (ng/ml)	106.9 (129.7)	164.4 (258.15)	**0.043**
Transferrin saturation (%)	17.13 (10.99)	14.95 (9.11)	**0.042**
CRP (mg/dl)	0.81 (0.66)	1.10 (0.83)	0.181
CRP >1 mg/dl (yes/no)	38/48	18/15	0.311
Kynurenine (µM)	7.23 (3.51)	9.15 (3.57)	**0.011**
Tryptophan (µM)	31.90 (18.58)	28.83 (18.68)	0.621
Kynurenine-to-tryptophan ratio	0.226 (0.119)	0.254 (0.158)	**0.024**

Values in bold font indicate statistically significant differences (P<0.05). HD, hemodialysis; HFrEF, heart failure with reduced ejection fraction; HF, heart failure; SGOT, serum glutamic oxaloacetic transaminase; SGPT, serum glutamic pyruvic transaminase; CRP, C-reactive protein.

**Table VIII:** Binary logistic regression analysis of factors associated with atrial fibrillation.

					95% CI for OR		
	B	S.E.	Sig.	OR	Lower	Upper	VIF
Age (per year)	0.092	0.035	**0.009**	1.096	1.023	1.175	1.363
Neutrophil-to-lymphocyte ratio	0.374	0.211	0.077	1.453	0.960	2.198	1.229
Hemoglobin (per g/dl)	-1.168	0.423	**0.006**	0.311	0.136	0.712	1.173
Phosphorus (per mg/dl)	-0.537	0.352	0.127	0.584	0.293	1.165	1.185
Ferritin (per ng/ml)	0.003	0.001	0.070	1.003	1.000	1.006	1.461
Transferrin saturation (%)	-0.072	0.041	0.076	0.930	0.859	1.007	1.521
Kynurenine (per µM)	0.316	0.153	**0.039**	1.372	1.016	1.853	1.465
Kynurenine-to-tryptophan ratio	4.225	2.783	0.129	68.401	0.292	16008	1.400

Model fit: Nagelkerke R^2^=0.498, AUC=0.871, model fit: X^2^=48.306, P<0.001. Values in bold font indicate statistically significant differences (P<0.05).

## Data Availability

The data generated in the present study may be requested from the corresponding author.
